# Hypoxia-inducible factor-1α activation in HPV-positive head and neck squamous cell carcinoma cell lines

**DOI:** 10.18632/oncotarget.20813

**Published:** 2017-09-11

**Authors:** Jennifer Knuth, Shachi J. Sharma, Nora Würdemann, Claudia Holler, Boyan K. Garvalov, Till Acker, Claus Wittekindt, Steffen Wagner, Jens P. Klussmann

**Affiliations:** ^1^ Department of Otorhinolaryngology, Head and Neck Surgery, University of Giessen, Giessen, Germany; ^2^ Institute of Neuropathology, University of Giessen, Giessen, Germany

**Keywords:** human papillomavirus (hpv), head and neck squamous cell carcinoma (hnscc), hypoxia, hypoxia inducible factor-1α (hif-1α), prolyl hydroxylase-domain protein 2 (phd2)

## Abstract

**Purpose:**

Human papillomavirus (HPV) is a causative agent for a rising number of head and neck squamous cell carcinomas (HNSCC), which are characterized by distinct tumor biology. Hypoxia inducible-factor (HIF) signaling influences initiation and progression of carcinogenesis and HPV oncoproteins have evolved to highjack cellular pathways for viral reproduction. Therefore, we investigated whether HPV activates HIF-1α expression in HNSCC.

**Experimental Technique:**

HPV-positive and -negative HNSCC cells were examined for adaptive responses to hypoxia. Expression of HIF-1α, prolyl hydroxylase-domain protein 2 (PHD2) and E-cadherin was analyzed by Western blotting, immunofluorescence (IF) microscopy and migration/wound healing assays.

**Results:**

HPV-positive HNSCC cells showed higher HIF-1α and PHD2 protein levels under normoxia and hypoxia. HIF-1α hydroxylation was reduced in HPV-positive HNSCC cell lines under PHD and proteasomal inhibition. *In vitro* wound healing assays showed impairment of migration and proliferation by HIF-1α pathway activation in HPV-negative cell lines only. In contrast, migration and proliferation in HPV-positive cell lines was impaired by HIF-1α specific siRNA.

**Conclusions:**

HPV-positive HNSCC cells show activation of the HIF pathway and adaptation to HIF-1α upregulation, representing potential therapeutic targets in this emerging tumor entity.

## INTRODUCTION

Human Papillomavirus (HPV)-associated head and neck squamous cell carcinoma (HNSCC) are considered to be a distinct tumor entity with clinical, pathological and molecular features that essentially differ from HPV-negative HNSCC [1–3]. Oncogenic activity of the viral proteins E6 and E7 is crucial in HPV-driven carcinogenesis [4]. Viral oncoproteins interact with many pivotal cellular regulators to induce metabolic remodeling “optimized” for viral replication and nutrient synthesis [5]. Interestingly, HPV oncoproteins are able to stabilize HIF-1α in different cell lines [6–8]. The group of hypoxia-inducible factors (HIF), especially HIF-1 plays a major role in supporting tumor metabolism and in cellular adaptation to hypoxic stress [9]. HIF-1 is a heterodimeric transcription factor consisting of an O_2_-sensitive HIF-1α subunit and a constitutively expressed HIF-1β subunit. The stability of the HIF-α (HIF-1α and HIF-2α) subunits is regulated by oxygen sensors of the prolyl hydroxylase domain (PHD) protein family. PHDs use molecular oxygen to hydroxylate specific prolyl residues within HIF-α, which creates a binding site for the von Hippel-Lindau (VHL) tumor-suppressor protein. The VHL tumor-suppressor protein is part of an E3 ubiquitin ligase complex that promotes rapid HIF-degradation [10]. Three PHDs exist (PHD1, 2 and 3), hydroxylating HIF-α subunits with different preferences, but PHD2 is considered to be the main regulator of HIF-1α setting its low steady-state levels under normoxia [11, 12]. Under hypoxia PHD activity is inhibited and HIF is stabilized.

HIF-1 activates the transcription of numerous downstream genes, regulating processes required for tumor cell survival and progression, such as glucose metabolism, cell proliferation, erythropoiesis, invasion, angiogenesis and apoptosis [9, 13]. E.g. it could be shown that HIF-1α is able to promote tumor progression and to induce epithelial-mesenchymal transition (EMT) of tumor cells to facilitate metastasis. This is characterized by upregulation of transcription effectors (e.g. Snail, Twist, ZEB1 and ZEB2), suppressing E-Cadherin expression [14].

HPV-related HNSCC have favorable outcome compared to HPV-negative forms of the disease, particular after ionizing irradiation [15]. This might be due to a more efficient ionization of oxygen in radiation therapy because of higher pO_2_ in those tumors. Consequently, a differentially regulated metabolic machinery in HPV-related oropharyngeal cancers could be expected and HPV oncoproteins have been shown to play a role in regulation of the metabolic machinery [5, 16]. Further, HIF-1α is also a crucial metabolic regulator in cancer cells and hypoxia is known to be related to radioresistance. Therefore, we investigated the HIF pathway in HPV-positive and -negative cancer cell lines under normoxia and hypoxia, to provide new insights for potential therapeutic strategies for HPV-associated HNSCC.

## RESULTS

### Increased HIF-1α protein levels in HPV-positive HNSCC cell lines under normoxia

**Figure 1:**
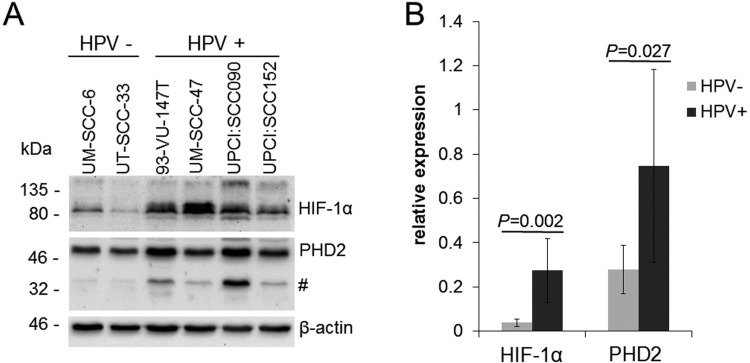
Increased HIF-1α protein levels in HPV-positive HNSCC cell lines under normoxia (**A**) Western blot of cell extracts of HPV-negative (UM-SCC-6 and UT-SCC-33) and HPV-positive HNSCC cell lines (UM-SCC-47, 93-VU-147T, UPCI:SCC090 and UPCI:SCC152) cultured under normoxia (21% O_2_). The HPV-positive HNSCC cell lines showed prominent protein bands reacting with the PHD2 antibody (#). (**B**) Quantification of basal HIF-1α and PHD2 protein levels detected by Western blotting normalized to β-actin expression. Data are represented as mean +/– SD (*n* = 3), P: *P*-value (*t*-test).

Protein expression of HIF-1α and PHD2 was evaluated in two HPV-negative and four HPV-positive HNSCC cell lines cultured under normoxic conditions (Figure 1). In the HPV-negative HNSCC cell lines, HIF-1α expression was low while strong bands corresponding to HIF-1α could be detected in cell extracts from HPV-positive HNSCC cell lines. Signal quantification showed that the mean HIF-1α protein levels were 7.2-fold higher in the HPV-positive than in the HPV-negative HNSCC cell lines (*P* = 0.002). Assessment of PHD2 expression under normoxia, revealed higher PHD2 protein levels in the HPV-positive HNSCC cell lines compared to the HPV-negative tumor cells (*P* = 0.027). HPV-positive cell lines showed potential PHD2 degradation products.

### Enhanced response to hypoxia in HPV-positive HNSCC cell lines

**Figure 2:**
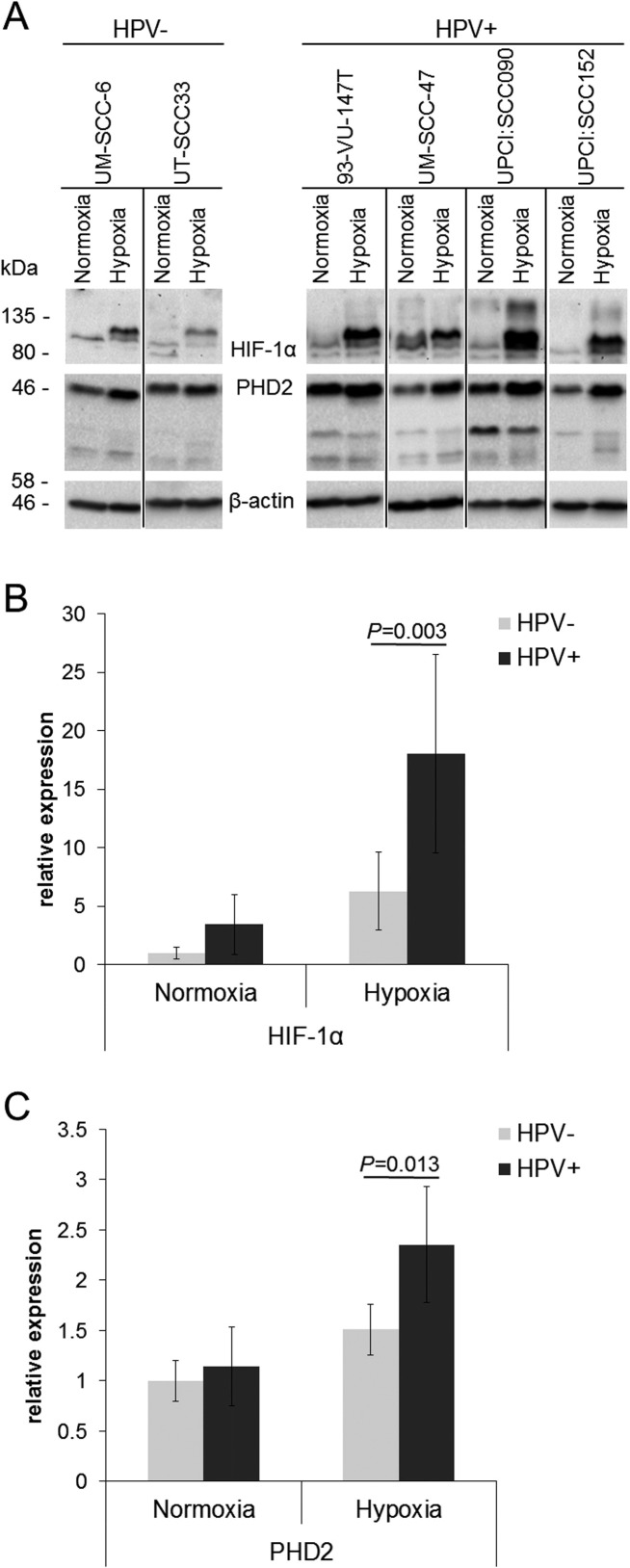
Enhanced response to hypoxia in HPV-positive HNSCC cell lines (**A**) Western blot of cell extracts of HPV-negative (UM-SCC-6 and UT-SCC-33) and HPV-positive cell lines (UM-SCC-47, 93-VU-147T, UPCI:SCC090 and UPCI:SCC152) cultured in hypoxia (1% O_2_) compared to normoxia. (**B**) Quantification of HIF-1α protein levels detected by Western blotting normalised to β-actin expression. (**C**) Quantification of PHD2 protein levels detected by Western blotting normalised to β-actin expression. The lowest HIF-1α and PHD2 values were set to 1 as reference for comparison. Data are represented as mean +/– SD (*n* = 3), P: *P*-value (*t*-test).

HIF-1α and PHD2 protein accumulation was evaluated in four HPV-positive and two HPV-negative cell lines under hypoxia (Figure 2). HPV-positive as well as HPV-negative HNSCC cell lines showed HIF-1α and PHD2 protein accumulation under hypoxia (Figure 2A). Signal quantification showed that the expression of HIF-1α increased under hypoxia in the HPV-positive HNSCC cell lines to a higher level compared to the HPV-negative cells (Figure 2B, relative expression 18.0 vs. 6.3, *P* = 0.003). The absolute increase in HIF-1α expression from normoxia to hypoxia was higher in HPV-positive compared to HPV-negative HNSCC cell lines (14.6 vs. 5.3 relative expression units, *P* = 0.008), although the relative increase compared to respective values under normoxia was similar (*P* = 0.472).

The expression of PHD2 also increased under hypoxia in the HPV-positive HNSCC cell lines to a higher level compared to the HPV-negative cells (Figure 2C, relative expression 2.4 vs. 1.5, *P* = 0.013). The absolute increase in PHD2 expression from normoxia to hypoxia was higher in HPV-positive compared to HPV-negative HNSCC cell lines (1.2 vs. 0.5 relative expression units, *P* = 0.003). In addition, the relative increase compared to respective values under normoxia was higher (2.1-fold vs. 1.5-fold, *P* = 0.001).

### Impaired HIF-1α hydroxylation in HPV-positive HNSCC cell lines

**Figure 3:**
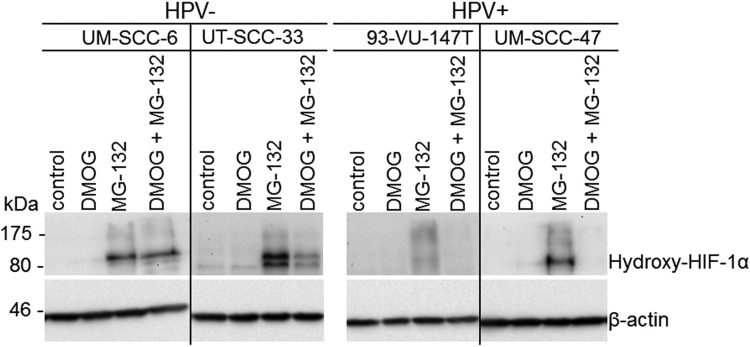
Impaired HIF-1α hydroxylation in HPV-positive HNSCC cell lines Western blot of cell extracts of HPV-negative (UM-SCC-6 and UT-SCC-33) and HPV-positive cell lines (UM-SCC47 and 93-VU-147T) cultured in DMOG and MG-132 (*n* = 3). In untreated controls and DMOG treated cells Hydroxy-HIF-1α levels could not be detected because of its rapid degradation. Dual inhibition of PHD-mediated hydroxylation by DMOG and proteasomal degradation of HIF-1α by MG-132 indicates low steady-state levels of Hydroxy-HIF-1α in HPV-positive compared to HPV-negative cell lines. Accumulation of protein reacting with Hydroxy-HIF-1α specific antibody, by inhibition of proteasomal HIF-1α degradation indicates functional hydroxylation of HIF-1α in all analyzed HNSCC cell lines.

Under normoxic conditions, HIF-1α is hydroxylated and rapidly degraded by the proteasome. Therefore, we analyzed its hydroxylated form after PHD inhibition with Dimethyloxalylglycine (DMOG) and blocking proteasomal degradation with MG-132 in two HPV-positive and two HPV-negative cell lines (Figure 3). Dual inhibition with DMOG and MG-132 shows the “steady-state” level of hydroxylated HIF-1α. Notably, both HPV-positive cell lines showed no detectable Hydroxy-HIF-1α protein levels under this condition, while in the HPV-negative HNSCC cells a strong Hydroxy-HIF-1α protein signal was observable. This is in line with the observed HIF-1α stabilization shown in Figure 1. After inhibition of proteasomal HIF-1α degradation, a strong Hydroxy-HIF-1α protein signal was obtained in the HPV-negative HNSCC cells. HPV-positive cell lines show less hydroxylation of HIF-1α indicated by a minor accumulation of protein reacting with Hydroxy- HIF-1α specific antibody.

### Enhanced upregulation of HIF-1α by chemical induction in HPV-positive HNSCC cell lines

**Figure 4:**
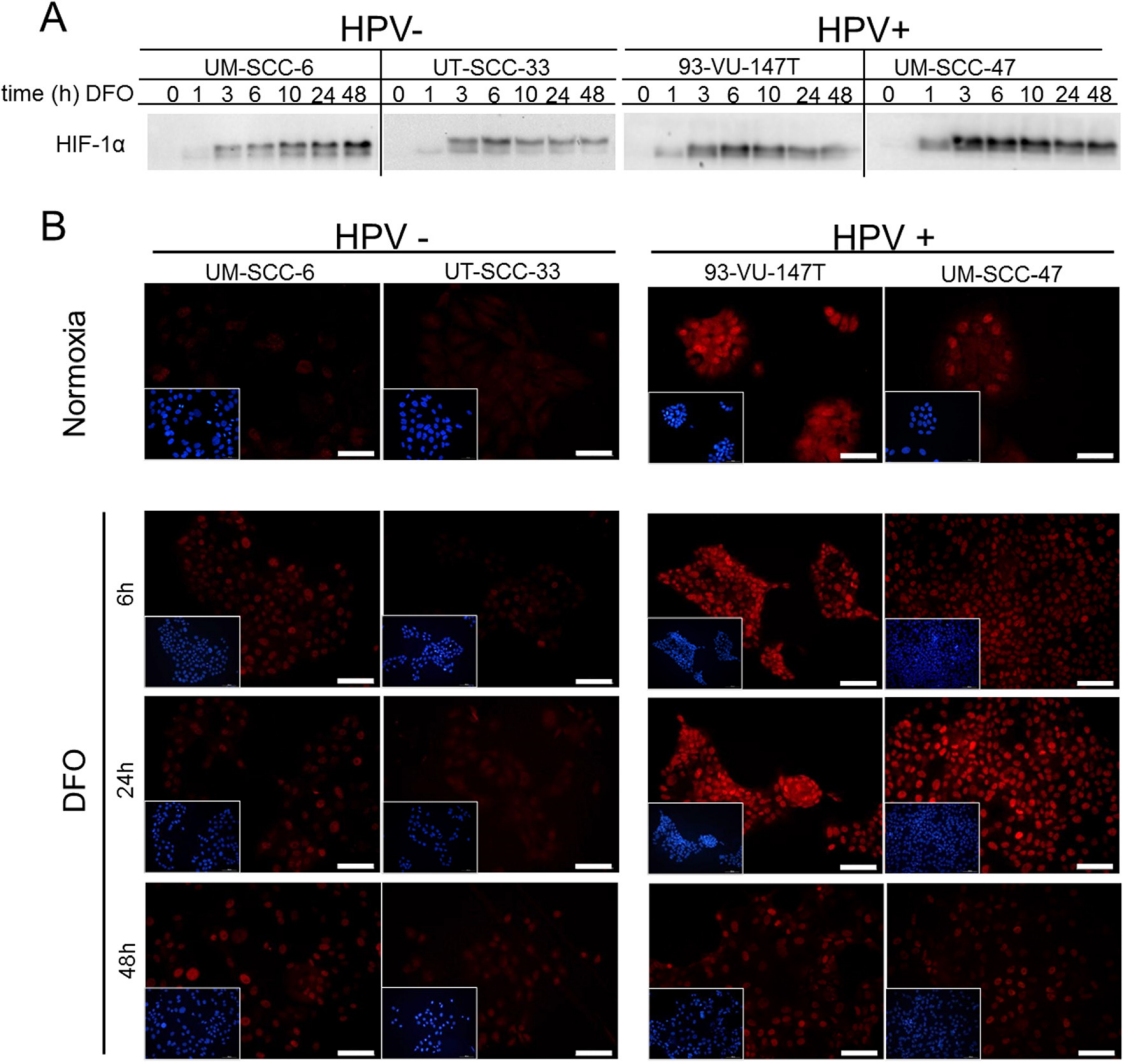
Enhanced upregulation of HIF-1α by chemical induction in HPV-positive HNSCC cell lines (**A**) Western blot of cell extracts of HPV-negative (UM-SCC-6 and UT-SCC-33) and HPV-positive HNSCC cell lines (UM-SCC47 and 93-VU-147T) showing HIF-1α protein expression at different time points after DFO addition. (**B**) Immunofluorescence staining of HIF-1α expression in the HPV-negative (left) and positive HNSCC cell lines (right) after incubation with DFO. Miniature views show nuclear staining (blue) of the same field which is shown by HIF-1α staining (red). In comparison to HPV-negative cells (UT-SCC-33; UM-SCC-6), HPV-positive cells (UM-SCC-47; 93-VU-147T) show increased basal HIF-1α expression under normoxia and enhanced upregulation of the nuclear HIF-1α signal by incubation with DFO. Normoxia: magnification 40x; bar represents 50 μm. Hypoxia: magnification 20x; bar represents 100 μm.

In addition to low levels of oxygen, treatment with the iron chelating agent deferoxamine (DFO) was also able to induce HIF-1α . Therefore, we tested whether the HIF-1α levels are consistently inducible through DFO over time. On average, all four cell lines showed an increase of HIF-1α signal in response to DFO-treatment after 6 hours (Figure 4A). To visualize HIF-1α expression and localization in HNSCC cell lines, immunofluorescence staining was performed (Figure 4B). HIF-1α protein expression and nuclear localization was observed in the HPV-positive HNSCC cells under normoxic culture conditions by immunofluorescence microscopy. The HPV-negative tumor cells had only faint staining for HIF-1α under normoxic conditions. Remarkably, enhanced HIF-1α signal and nuclear localization after DFO-treatment was shown in the HPV-positive HNSCC cells compared to the HPV-negative tumor cells.

### Chemically induced hypoxia impairs the migration of HPV-negative cell lines while HPV-positive cell lines are influenced by HIF-1α specific siRNA

**Figure 5:**
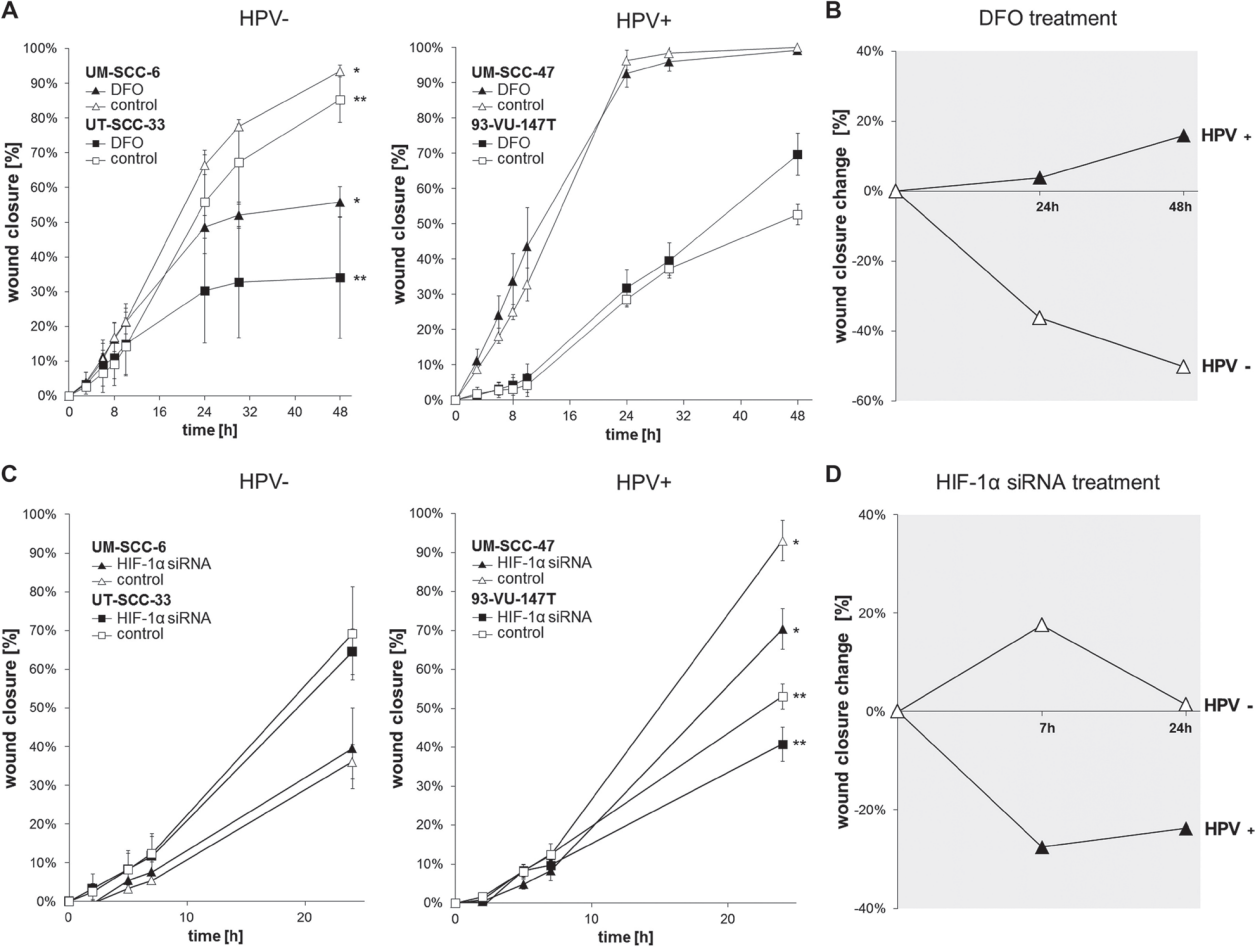
Chemically induced hypoxia impairs the migration of HPV-negative while HPV-positive cell lines are influenced via HIF-1α inhibition by siRNA (**A**) Scratch assay showing uninfluenced migration- and wound healing potential in HPV-positive cell lines and significant impairment of HPV-negative cell lines (*UM-SCC-6: *P* < 0.001, ** UT-SCC-33: *P* = 0.018) under chemically induced hypoxia (*t*-test (48 h): DFO vs. control). Data are represented as mean +/– SD (*n* = 3). (**B**) Summarizing the data, HPV-positive cell lines showed uninfluenced or even better wound closure change after HIF-1α activation by DFO treatment while HPV-negative cell lines showed worse wound closure. Data are represented as percentage increase or decrease in wound closure change (treatment/control). (**C**) Treatment with HIF-1α siRNA showed significantly impaired migration and proliferation of the HPV-positive cell lines (*UM-SCC-47: *P* = 0.012, **93-VU-147T: *P* = 0.034), but uninfluenced HPV-negative cell lines (*t*-test (24 h): HIF-1α siRNA vs. control). Data are represented as mean +/– SD (*n* = 3). D: HPV-positive cell lines showed inferior wound closure change with HIF-1α inhibition by siRNA while HPV-negative cells showed uninfluenced wound closure. Data are represented as percentage increase or decrease in wound closure change (treatment/control).

In order to investigate the difference between HPV-positive and HPV-negative HNSCC cell lines in cellular adaptation to hypoxia, we performed *in vitro* wound healing assays with or without chemical induction of HIF-signaling by DFO (Figure 5A) and HIF-1α inhibition by siRNA (Figure 5C). The results showed DFO related impairment of migration and proliferation in HPV-negative cell lines only (Figure 5A, UM-SCC-6: *P* < 0.001, UT-SCC-33: *P* = 0.018). Remarkably, the migration- and proliferation was not reduced in the HPV-positive HNSCC cell lines by chemically induced hypoxia. On the other hand, HIF-1α inhibition by siRNA showed impaired migration and proliferation in HPV-positive cell lines (Figure 5C, UM-SCC-47: *P* = 0.012, 93-VU-147T: *P* = 0.034), but not in HPV-negative cell lines. As shown in the graphs summarizing the data of Figure 5A and 5C, HPV-positive and -negative cell lines showed contrary effects in wound closure change (Figure 5B, 5D). HPV-positive cell lines showed uninfluenced or even enhanced wound closure after HIF-1α activation by DFO treatment (Figure 5B), but inferior wound closure with HIF-1α inhibition by siRNA (Figure 5D) compared to the control. In contrast, HPV-negative cells showed uninfluenced wound closure by siRNA treatment (Figure 5D) and worse wound closure by DFO treatment (Figure 5B) compared to the control.

### Reduction of membranous E-cadherin staining in HPV-positive HNSCC cell lines by HIF-1α stabilization

**Figure 6:**
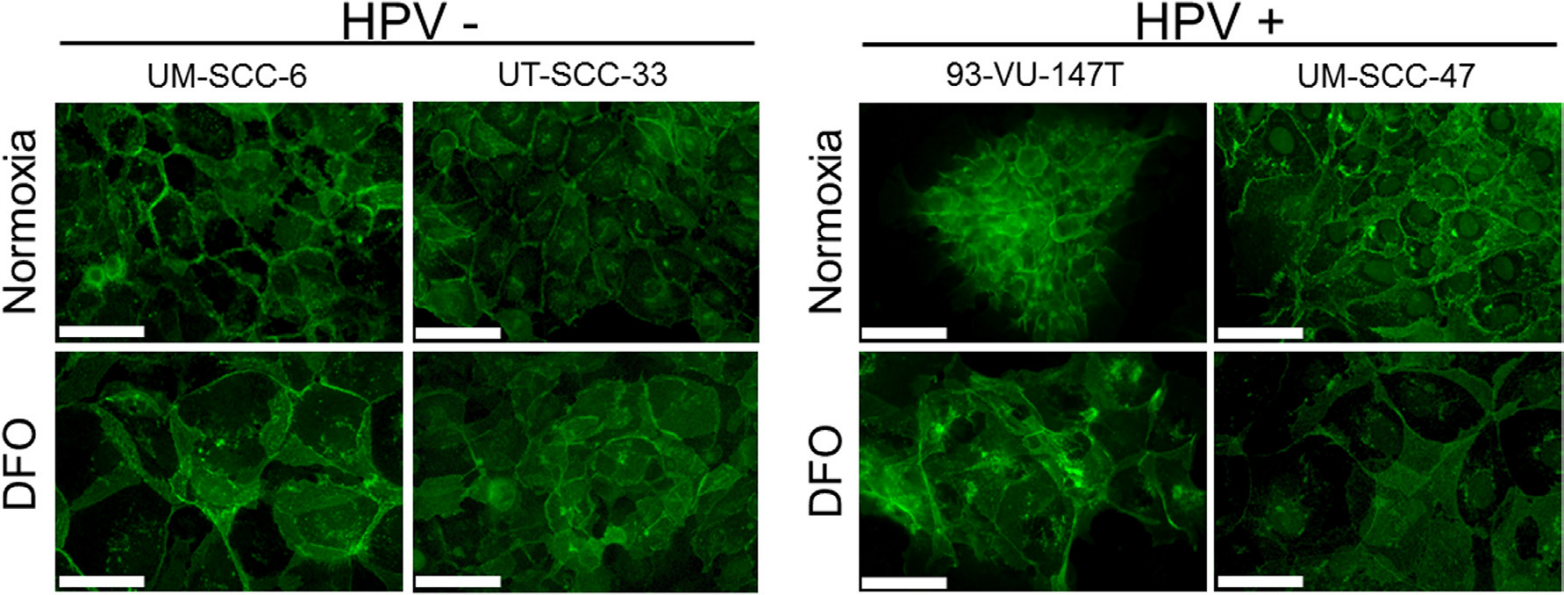
Reduced membranous E-cadherin staining in HPV-positive HNSCC cell lines Immunofluorescence staining of E-cadherin expression (green) in the HPV-negative (left) and positive HNSCC cell lines (right) under normoxia and after incubation with DFO for 48 hours. In comparison to HPV-negative cells (UM-SCC-6, UT-SCC-33), the HPV-positive cell lines (93-VU-147T, UM-SCC-47) showed reduced membranous E-cadherin staining after incubation with DFO. Normoxia and hypoxia: magnification 40×; bar represents 50 μm.

Under normoxia, all cell lines presented with a membranous staining pattern of E-cadherin (Figure 6). Under hypoxia pathway activation by DFO treatment, reduced membranous E-cadherin staining in HPV-positive compared to the HPV-negative tumor cells was observed.

## DISCUSSION

For HNSCC, we showed significantly higher HIF-1α protein levels under normoxia in HPV-positive compared to HPV-negative cell lines. In cervical cancer cell lines and HPV-transfected human foreskin keratinocytes, stabilization of HIF-1α protein levels under normoxia was not reported [8]. In line with our results, HIF-1α protein accumulation under normoxic conditions was found in HPV16 E6 and E7 transfected cell lines. But it could not be shown that HPV16 E6 or E7 effects HIF-1α protein stabilization or mRNA levels, suggesting an altered regulation at the translational level *in vitro* [17, 18]. Data from clinical samples is contradictory. No association between HPV-status and HIF-1α was shown in Oral Squamous Cell Carcinoma (OSCC) [19, 20]. In line with our results, a significantly positive correlation between HIF-1α and HPV16 E7 labeling indexes in OSCC could be shown [21], suggesting a positive association between HIF-1α and HPV oncoprotein expression. Further, activated hypoxic signaling in HNSCC, especially in HPV-related tumors was suggested because of highly expressed hypoxia-associated proteins [22].

The main regulator of HIF-1α is PHD2. Hydroxylation of HIF-1α by PHD2 and further ubiquitination initiated by binding to VHL-protein causes proteasomal degradation under normoxia. Inhibition of PHD2, but not PHD1 or PHD3 by siRNA is sufficient to upregulate HIF-1α under normoxic conditions, which has been observed in various cancer and primary cell types [12]. We found differences between HPV-positive and -negative cell lines with respect to the regulation of HIF-1α and PHD2 under normoxia and elevated HIF-1α levels might be explained by HPV oncoprotein induced reduction of PHD2-activity. Recent data also suggested an inhibition of HIF-1α hydroxylation via binding of viral proteins to PHDs, preventing proteasomal degradation and resulting in HIF-1α stabilization in EBV-transformed B-cells [23]. In line with this, we observed a reduced steady-state level of HIF-1α hydroxylation in the HPV-positive cell lines by experimental inhibition of PHDs and the proteasome. In conclusion, reduced HIF-1a hydroxylation and degradation by HPV oncoprotein mediated PHD inhibition might explain the elevated HIF-1α protein levels under normoxia in our experiments.

Under hypoxia, we show upregulation of HIF-1α in all HNSCC cell lines, implying a functional hypoxia response pathway. However, the relative hypoxic expression signal and absolute protein increase was higher in the HPV-positive cell lines. Recently, it has been shown that HIF-1α protein accumulates in HPV-positive and -negative HNSCC cell lines under hypoxia [24], but no elevated accumulation of HIF-1α protein levels in the HPV-positive cell lines was detected under normoxic conditions [25]. In accordance with our investigations, earlier studies have shown HIF-1α stabilization by HPV under hypoxic conditions (chemically induced) in HPV E6/E7 oncoprotein transfected cells or cervical cell lines [6, 8, 26].

In line with established regulatory mechanisms [27–33], elevated HIF-1α levels in HPV-positive cell lines were associated with a significant increase of PHD2 levels under hypoxia in our study. We conclude that HPV oncoprotein induced HIF-1α activation under normoxic conditions occurs via inhibition of PHD2 activity and is further enhanced by decreasing oxygen supply.

To determine the functionality of HIF-1α in head and neck cancer cells, we investigated its cellular localization. Nuclear localization implies a functional HIF-1α protein that can activate its target genes [23]. In our study, a more intense fluorescence signal in HIF-1α positive stained nuclei from HPV-positive cell lines compared to HPV-negative cell lines was detected under normoxia. Additionally, an increase of nuclear HIF-1α accumulation under DFO treatment was observable. Both findings suggest a functional HIF-1α in HPV-associated HNSCC. HIF-1α activation could result in a ‘Warburg-like effect’ in HPV-related tumors under normoxia influencing tumor growth and improving cell survival [28]. In addition, HIF-1α can stimulate angiogenesis [33], which could contribute higher radiosensitivity [3, 15] due to improved oxygen supply in HPV-associated tumors.

Both HIF-1α suppression and overexpression highly influence the growth of hypoxic tumors [34–36]. We found different adaptive proliferation and migration abilities under hypoxia pathway activation and HIF-1α inhibition by siRNA in the investigated cell lines, indicating high hypoxic adaptation in the HPV-positive cell lines. This is in concordance with the highly induced HIF-1α levels in those cell lines.

Additionally, we found degradation and a loss of membranous E-cadherin after DFO-treatment in the HPV-positive cell lines. Promoted proliferation, migration and invasion is most likely due to impaired-cadherin mediated cell-cell adhesion or E-cadherin ectodomain shedding [37, 38]. Thus, our results are compatible with HIF-1α mediated E-cadherin shedding in HPV-positive cell lines, which could activate EMT [14, 39]. This and suppressed expression of E-Cadherin might explain the metastasizing phenotype in HPV-associated HNSCC [40].

In conclusion, the impaired HIF-1α hydroxylation and increased HIF-1α levels in HPV-positive HNSCC cell lines we observed could be clinically relevant for those patients with tumors of high metastasizing potential. Re-establishing of HIF-1α degradation by interfering with the HPV-HIF-1α interaction might suppress tumor growth and metastasis.

## MATERIALS AND METHODS

### Cell lines and culture conditions

Cell lines were cultured in RPMI1640 medium supplemented with 10% fetal bovine serum (FBS), 2 mM L-glutamine, 1% non-essential amino acids and 0.1% gentamicin and maintained in a humidified atmosphere (5% CO_2_) at 37°C under normoxic (21% O_2_) or hypoxic conditions (1% O_2_ for 24 hours). Chemical induction of hypoxia was done by adding 150 μM deferoxamine (DFO, Sigma-Aldrich, St. Louis, USA) for 1–48 hours (time series experiment). Cell line UM-SCC-47 (HPV-16 positive) was provided by T.E. Carey, University of Michigan, United States [41], UM-SCC-6 (HPV-negative), was purchased from Merck KGaA, Darmstadt [41] cell line 93-VU-147T (HPV-16 positive) was provided by J.P. de Winter, VU Medical Centre, Amsterdam, Netherlands [42] and UT-SCC-33 (HPV-negative) was provided by R.A. Grenman, Turku University, Finland [43], cell line UPCI:SCC090 (HPV-16 positive) and UPCI:SCC152 (HPV-positive) were provided by S. M. Gollin, University of Pittsburgh, United States [44, 45].

### Western blot

Cell extracts were generated after washing cells (once with 1xPBS) by using lysis buffer (1 mM Tris/HCl pH 7.5, 2% SDS, 2mM EDTA, 20 mM NaF) and sonication (Ultrasonic-processor UIS250L, Hielscher Ultrasonics GmbH, Teltow, Germany; 100%, cycle 0.5). Cleared extracts were obtained by centrifugation (21100 × g for 10 min at 20°C). Protein concentration was determined using the Pierce™ BCA Protein Assay Kit (Thermo Scientific™, Rockford, USA) and 30 or 40 μg of total protein were separated on 10% and 12% polyacrylamide (PAA) SDS gels and transferred to nitrocellulose membrane (Protran, Whatman, Sigma-Aldrich, St. Louis, USA). Primary antibodies against HIF-1α (clone 54, BD Transduction laboratories™, Germany; 1/600 diluted in 2% BSA/TBST), the hydroxylated form of HIF-1α (Hydroxy-HIF-1α (Pro564) (D43B5) Cell Signaling Technology^®^, Danvers, Massachusetts, USA; 1/600 and PHD2/EGLN1 (Cell Signaling Technology^®^, Danvers, Massachusetts, USA; 1/1000 diluted in 2% BSA/TBST) were used with respective horseradish peroxidase (HRP) conjugated secondary antibodies. Immun-Star™ WesternC^™^ chemiluminescent substrate (Bio-Rad Laboratories, Munich, Germany) was used to visualize labeled protein bands in a ChemiDoc^™^ XRS+ Imager (Bio-Rad Laboratories, Munich, Germany). ColorPlus^™^ Prestained protein marker (New England Biolabs^®^_GmbH_, Frankfurt am Main, Germany), Broad Range (7-175kDa) was used to estimate the molecular weight of protein bands.

HIF-1α and PHD2 protein expression was quantified and normalized to actin signals (β-actin mouse mAb; Cell Signaling Technology^®^, Danvers, Massachusetts, USA; 1/10000 diluted in 5% milk/TBST) on each blot (*n* = 3).

### Immunofluorescence

Cells were seeded on coverslips and incubated until they reached approximately 80% confluences in the presence or absence of DFO (150 μM for 24 hours). Fixed cells (with 4% formaldehyde (FA) and washed three times with PBS/100 mM glycine) were probed with primary antibodies against HIF-1α (clone 541/150 diluted in 1% BSA/TBST). Anti-mouse IgG (whole molecule) F(ab′)2 fragment–Cy3 antibody (Sigma-Aldrich, St. Louis, USA; 1/800 diluted in 1% BSA/TBST) was used to visualize the primary HIF-1α antibody. Alexa Fluor^®^ 488 Mouse conjugated anti E-cadherin antibody (BD Transduction laboratories^™^, Germany; 1/150 diluted in 1% BSA/TBST) was used for staining the transmembrane glycoprotein E-cadherin. Nuclear staining was performed with Roti^®^-Mount FluorCare DAPI (Carl Roth, GmbH + Co. KG, Karlsruhe, Germany; 1:1). Immunofluorescence staining was evaluated using Leica DM2500 and Leica Application Suite X (LAS X) (Leica Microsystems, Wetzlar, Germany).

### Functional assays

Migration- and proliferation potential was determined in “scratch” (wound healing) assays. Cells were seeded in 3.5 or 6 cm petri dishes. After reaching 100% confluence, a cross-shaped “scratch” was created using a 10 μl pipette tip. Detached cells were removed by washing tree times with 1xPBS. Cells were cultured in RPMI1640 for 48 hours with or without 150 μM DFO or 10 nM HIF-1α siRNA (s6541, Thermo Fisher Scientific Inc., Waltham, MA USA 02451). Images were taken with an inverse microscope (Leica DMI3000B, Leica Microsystems, Wetzlar, Germany) at time points: 0, 3, 6, 8, 10, 24 and 48 h under DFO treatment or 0, 2, 5, 7, 24 h under siRNA treatment after scratching. Analysis of the wound healing area was done with image processing program (ImageJ 1.49). Each experiment was done three times and six images or three in the case of siRNA experiments were analyzed for each time point.

### Proteasomal blocking and PHD inhibition

Under normoxic conditions HIF-1α is usually hydroxylated and degraded very fast by the proteasome, therefore we assayed its hydroxylated form after inhibition of PHDs with Dimethyloxalylglycine (DMOG) (1.5 mM) and blocking proteasomal degradation with MG-132 (40 μM). Cells were grown in 3.5 cm petri dishes until reaching 60–70% confluences. After replacing the culture medium, cells were cultured for 10 hours with the membrane-permeable proteasome inhibitor MG-132 and DMOG, both from Sigma-Aldrich, St. Louis, USA. Protein extraction and western blotting was performed as described above. Untreated cells were used as control.

## Abbreviations

DFO: Deferoxamine
DMOG: Dimethyloxalylglycine
EMT: Epithelial-mesenchymal transition
FA: Formaldehyde
FBS: fetal bovine serum
HIF: Hypoxia inducible-factor
HIF-1α: Hypoxia inducible factor-1α
HNSCC: Head and Neck Squamous Cell Carcinoma
HRP: Horseradish peroxidase
HPV: Human Papillomavirus
Hydroxy-HIF-1α: Hydroxylated form of HIF-1α
IF: Immunofluorescence
MG-132: Membrane-permeable proteasome inhibitor
PAA: Polyacrylamide
PHD2: Prolyl hydroxylase-domain protein 2.

## References

[1] Kreimer AR, Clifford GM, Boyle P, Franceschi S (2005). Human papillomavirus types in head and neck squamous cell carcinomas worldwide: a systematic review. Cancer Epidemiol Biomarkers Prev.

[2] Leemans CR, Braakhuis BJ, Brakenhoff RH (2011). The molecular biology of head and neck cancer. Nat Rev Cancer.

[3] Lindquist D, Romanitan M, Hammarstedt L, Nasman A, Dahlstrand H, Lindholm J, Onelov L, Ramqvist T, Ye W, Munck-Wikland E, Dalianis T (2007). Human papillomavirus is a favourable prognostic factor in tonsillar cancer and its oncogenic role is supported by the expression of E6 and E7. Mol Oncol.

[4] zur Hausen H (2002). Papillomaviruses and cancer: from basic studies to clinical application. Nat Rev Cancer.

[5] Noch E, Khalili K (2012). Oncogenic viruses and tumor glucose metabolism: like kids in a candy store. Mol Cancer Ther.

[6] Bodily JM, Mehta KP, Laimins LA (2011). Human papillomavirus E7 enhances hypoxia-inducible factor 1-mediated transcription by inhibiting binding of histone deacetylases. Cancer Res.

[7] Cuninghame S, Jackson R, Zehbe I (2014). Hypoxia-inducible factor 1 and its role in viral carcinogenesis. Virology.

[8] Nakamura M, Bodily JM, Beglin M, Kyo S, Inoue M, Laimins LA (2009). Hypoxia-specific stabilization of HIF-1alpha by human papillomaviruses. Virology.

[9] Masoud GN, Li W (2015). HIF-1alpha pathway: role, regulation and intervention for cancer therapy. Acta Pharm Sin B.

[10] Semenza GL (2014). Oxygen sensing, hypoxia-inducible factors, and disease pathophysiology. Annu Rev Pathol.

[11] Berra E, Benizri E, Ginouves A, Volmat V, Roux D, Pouyssegur J (2003). HIF prolyl-hydroxylase 2 is the key oxygen sensor setting low steady-state levels of HIF-1alpha in normoxia. Embo J.

[12] Karuppagounder SS, Ratan RR (2012). Hypoxia-inducible factor prolyl hydroxylase inhibition: robust new target or another big bust for stroke therapeutics?. J Cereb Blood Flow Metab.

[13] Bardos JI, Ashcroft M (2005). Negative and positive regulation of HIF-1: a complex network. Biochim Biophys Acta.

[14] Zhang Q, Bai X, Chen W, Ma T, Hu Q, Liang C, Xie S, Chen C, Hu L, Xu S, Liang T (2013). Wnt/beta-catenin signaling enhances hypoxia-induced epithelial-mesenchymal transition in hepatocellular carcinoma via crosstalk with hif-1alpha signaling. Carcinogenesis.

[15] Arenz A, Ziemann F, Mayer C, Wittig A, Dreffke K, Preising S, Wagner S, Klussmann JP, Engenhart-Cabillic R, Wittekindt C (2014). Increased radiosensitivity of HPV-positive head and neck cancer cell lines due to cell cycle dysregulation and induction of apoptosis. Strahlenther Onkol.

[16] Mazurek S, Zwerschke W, Jansen-Durr P, Eigenbrodt E (2001). Effects of the human papilloma virus HPV-16 E7 oncoprotein on glycolysis and glutaminolysis: role of pyruvate kinase type M2 and the glycolytic-enzyme complex. Biochem J.

[17] Li G, He L, Zhang E, Shi J, Zhang Q, Le AD Zhou K, Tang X (2011). Overexpression of human papillomavirus (HPV) type 16 oncoproteins promotes angiogenesis via enhancing HIF-1alpha and VEGF expression in non-small cell lung cancer cells. Cancer Lett.

[18] Tang X, Zhang Q, Nishitani J, Brown J, Shi S, Le AD (2007). Overexpression of human papillomavirus type 16 oncoproteins enhances hypoxia-inducible factor 1 alpha protein accumulation and vascular endothelial growth factor expression in human cervical carcinoma cells. Clin Cancer Res.

[19] Jo S, Juhasz A, Zhang K, Ruel C, Loera S, Wilczynski SP, Yen Y, Liu X, Ellenhorn J, Lim D, Paz B, Somlo G, Vora N (2009). Human papillomavirus infection as a prognostic factor in oropharyngeal squamous cell carcinomas treated in a prospective phase II clinical trial. Anticancer Res.

[20] Hong A, Zhang M, Veillard AS, Jahanbani J, Lee CS, Jones D, Harnett G, Clark J, Elliott M, Milross C, Rose B (2013). The prognostic significance of hypoxia inducing factor 1-alpha in oropharyngeal cancer in relation to human papillomavirus status. Oral Oncol.

[21] Rodolico V, Arancio W, Amato MC, Aragona F, Cappello F, Di Fede O, Pannone G, Campisi G (2011). Hypoxia inducible factor-1 alpha expression is increased in infected positive HPV16 DNA oral squamous cell carcinoma and positively associated with HPV16 E7 oncoprotein. Infect Agent Cancer.

[22] Choi HG, Kim JS, Kim KH, Sung MW, Choe JY, Kim JE, Jung YH (2015). Expression of hypoxic signaling markers in head and neck squamous cell carcinoma and its clinical significance. Eur Arch Otorhinolaryngol.

[23] Darekar S, Georgiou K, Yurchenko M, Yenamandra SP, Chachami G, Simos G, Klein G, Kashuba E (2012). Epstein-Barr virus immortalization of human B-cells leads to stabilization of hypoxia-induced factor 1 alpha, congruent with the Warburg effect. PLoS One.

[24] Hanns E, Job S, Coliat P, Wasylyk C, Ramolu L, Pencreach E, Suarez-Carmona M, Herfs M, Ledrappier S, Macabre C, Abecassis J, Wasylyk B, Jung AC (2015). Human Papillomavirus-related tumours of the oropharynx display a lower tumour hypoxia signature. Oral Oncol.

[25] Jung YS, Najy AJ, Huang W, Sethi S, Snyder M, Sakr W, Dyson G, Huttemann M, Lee I, Ali-Fehmi R, Franceschi S, Struijk L, Kim HE (2017). HPV-associated differential regulation of tumor metabolism in oropharyngeal head and neck cancer. Oncotarget.

[26] Guo Y, Meng X, Ma J, Zheng Y, Wang Q, Wang Y, Shang H (2014). Human papillomavirus 16 E6 contributes HIF-1alpha induced Warburg effect by attenuating the VHL-HIF-1alpha interaction. Int J Mol Sci.

[27] D’Angelo G, Duplan E, Boyer N, Vigne P, Frelin C (2003). Hypoxia up-regulates prolyl hydroxylase activity: a feedback mechanism that limits HIF-1 responses during reoxygenation. J Biol Chem.

[28] Denko NC (2008). Hypoxia, HIF1 and glucose metabolism in the solid tumour. Nat Rev Cancer.

[29] Ginouves A, Ilc K, Macias N, Pouyssegur J, Berra E (2008). PHDs overactivation during chronic hypoxia “desensitizes” HIFalpha and protects cells from necrosis. Proc Natl Acad Sci USA.

[30] Henze AT, Acker T (2010). Feedback regulators of hypoxia-inducible factors and their role in cancer biology. Cell Cycle.

[31] Henze AT, Riedel J, Diem T, Wenner J, Flamme I, Pouyseggur J, Plate KH, Acker T (2010). Prolyl hydroxylases 2 and 3 act in gliomas as protective negative feedback regulators of hypoxia-inducible factors. Cancer Res.

[32] Marxsen JH, Stengel P, Doege K, Heikkinen P, Jokilehto T, Wagner T, Jelkmann W, Jaakkola P, Metzen E (2004). Hypoxia-inducible factor-1 (HIF-1) promotes its degradation by induction of HIF-alpha-prolyl-4-hydroxylases. Biochem J.

[33] Semenza GL (2007). HIF-1 mediates the Warburg effect in clear cell renal carcinoma. J Bioenerg Biomembr.

[34] Lim JH, Park JW, Kim MS, Park SK, Johnson RS, Chun YS (2006). Bafilomycin induces the p21-mediated growth inhibition of cancer cells under hypoxic conditions by expressing hypoxia-inducible factor-1alpha. Mol Pharmacol.

[35] Ryan HE, Poloni M, McNulty W, Elson D, Gassmann M, Arbeit JM, Johnson RS (2000). Hypoxia-inducible factor-1alpha is a positive factor in solid tumor growth. Cancer Res.

[36] Kung AL, Wang S, Klco JM, Kaelin WG, Livingston DM (2000). Suppression of tumor growth through disruption of hypoxia-inducible transcription. Nat Med.

[37] Klucky B, Mueller R, Vogt I, Teurich S, Hartenstein B, Breuhahn K, Flechtenmacher C, Angel P, Hess J (2007). Kallikrein 6 induces E-cadherin shedding and promotes cell proliferation, migration, and invasion. Cancer Res.

[38] Reiss K, Ludwig A, Saftig P (2006). Breaking up the tie: disintegrin-like metalloproteinases as regulators of cell migration in inflammation and invasion. Pharmacol Ther.

[39] Rankin EB, Giaccia AJ (2016). Hypoxic control of metastasis. Science.

[40] Huang CG, Lee LA, Tsao KC, Liao CT, Yang LY, Kang CJ, Chang KP, Huang SF, Chen IH, Yang SL, Lee LY, Hsueh C, Chen TC (2014). Human papillomavirus 16/18 E7 viral loads predict distant metastasis in oral cavity squamous cell carcinoma. J Clin Virol.

[41] Krause CJ, Carey TE, Ott RW, Hurbis C, McClatchey KD, Regezi JA (1981). Human squamous cell carcinoma. Establishment and characterization of new permanent cell lines. Arch Otolaryngol.

[42] Steenbergen RD, Hermsen MA, Walboomers JM, Joenje H, Arwert F, Meijer CJ, Snijders PJ (1995). Integrated human papillomavirus type 16 and loss of heterozygosity at 11q22 and 18q21 in an oral carcinoma and its derivative cell line. Cancer Res.

[43] Lansford C, Grenman R, Bier H, Somers KD, Kim SY, Whiteside TL, Clayman GL, Welkoborsky HJ, Carey TE, J PBM (1999). Head and neck cancers. Human cell culture cancer cell lines part 2.

[44] White JS, Weissfeld JL, Ragin CC, Rossie KM, Martin CL, Shuster M, Ishwad CS, Law JC, Myers EN, Johnson JT, Gollin SM (2007). The influence of clinical and demographic risk factors on the establishment of head and neck squamous cell carcinoma cell lines. Oral Oncol.

[45] Martin CL, Reshmi SC, Ried T, Gottberg W, Wilson JW, Reddy JK, Khanna P, Johnson JT, Myers EN, Gollin SM (2008). Chromosomal imbalances in oral squamous cell carcinoma: examination of 31 cell lines and review of the literature. Oral Oncol.

